# 

**Published:** 1892-08

**Authors:** 


Entered at the Post Office at Austin, Texas, as Second-Class Mail Matter.

VOL. VIII. AUGUST, 1892. No. 2.

### DANIEL'S TEXAS MEDICAL JOURNAL

SIT LUX

Subscription, $2.00 a Year, in Advance.—Single Copies, Twenty-five Cents.

F. E. DANIEL, M. D., Editor and Publisher, AUSTIN, TEXAS.

